# Integrative polygenic analysis of the protective effects of fatty acid metabolism on disease as modified by obesity

**DOI:** 10.3389/fnut.2023.1308622

**Published:** 2024-01-15

**Authors:** Courtney Astore, Greg Gibson

**Affiliations:** Center for Integrative Genomics and School of Biological Sciences, Georgia Institute of Technology, Atlanta, GA, United States

**Keywords:** fatty acid metabolites, PheWAS, obesity, canalization, polygenic scores, Mendelian randomization

## Abstract

Dysregulation of fatty acid metabolites can play a crucial role in the progression of complex diseases, such as cardiovascular disease, digestive diseases, and metabolic diseases. Metabolites can have either protective or risk effects on a disease; however, the details of such associations remain contentious. In this study, we demonstrate an integrative PheWAS approach to establish high confidence, causally suggestive of metabolite–disease associations for three fatty acid metabolites, namely, omega-3 fatty acids, omega-6 fatty acids, and docosahexaenoic acid, for 1,254 disease endpoints. Metabolite–disease associations were established if there was a concordant direction of effect and significance for metabolite level and genetic risk score for the metabolite. There was enrichment for metabolite associations with diseases of the respiratory system for omega-3 fatty acids, diseases of the circulatory system and endocrine system for omega-6 fatty acids, and diseases of the digestive system for docosahexaenoic acid. Upon performing Mendelian randomization on a subset of the outcomes, we identified 3, 6, and 15 significant diseases associated with omega-3 fatty acids, omega-6 fatty acids, and docosahexaenoic acid, respectively. We then demonstrate a class of prevalence-risk relationships indicative of (de)canalization of disease under high and low fatty acid metabolite levels. Finally, we show that the interaction between the metabolites and obesity demonstrates that the degree of protection afforded by fatty acid metabolites is strongly modulated by underlying metabolic health. This study evaluated the disease architectures of three polyunsaturated fatty acids (PUFAs), which were validated by several PheWAS modes of support. Our results not only highlight specific diseases associated with each metabolite but also disease group enrichments. In addition, we demonstrate an integrative PheWAS methodology that can be applied to other components of the human metabolome or other traits of interest. The results of this study can be used as an atlas to cross-compare genetic with non-genetic disease associations for the three PUFAs investigated. The findings can be explored through our R shiny app at https://pufa.biosci.gatech.edu.

## 1 Introduction

The human body contains thousands of circulating metabolites that are critical for maintaining homeostasis across several vital pathways. Because metabolites serve as mediators supporting energy metabolism for metabolic pathways, they can be evaluated as possible biomarkers and therapeutic targets of disease (1, 2). Additionally, metabolites can also be referred to as endophenotypes, which serve as intermediate phenotypes affected by environmental exposures that influence the risk of diseases (3). Although it is evident that dysregulation of metabolites can yield disease, comprehensive significant exposure-outcome associations are yet to be evaluated.

Essential polyunsaturated fatty acids (PUFAs) have long been investigated for their roles in development and disease progression. It is generally acknowledged that omega-3 fatty acids, particularly α-linolenic (ALA), eicosapentanoic (EPA), and docosahexanoic (DHA) acids, are anti-inflammatory and, to some extent, protective with respect to multiple classes of disease, including autoimmune, cardiovascular, neuropsychiatric disease, and cancer (4–7). However, recent meta-analyses of the effects of dietary supplementation have failed to find significant replicated benefits (8–10). Consequently, support for widespread adoption by healthy individuals is lacking, and conversely, there is some concern that they may be harmful in excess, for example, inducing gastrointestinal bleeding or atrial fibrillation (*cf.* 11, 12). Alternatively, it is also clear that Western diets have recently greatly elevated the ratio of omega-6 to omega-3 PUFAs, and since the former increases the production of inflammatory cytokines of the prostaglandin and eicosanoid families, a detrimental effect of modern diet is implicated. Plant oils are a primary source of ALA, which can be elongated in the body to generate DHA, which is also enriched in certain fish oils. PUFAs also indirectly regulate disease risk through modulation of the gut microbiome (13, 14), which, in turn, influences immune and mental health.

Historically, relationships between PUFAs and disease have been explored through correlational disease-specific studies, asking whether there is a mean difference between the abundance of a specific metabolite and the condition. Such studies do not allow inference of causation, since correlations may be due to confounders, with which the metabolite is itself correlated, or differences may arise after the onset of the pathology. Genetic associations bring us a step closer to causation, as a difference in mean polygenic score that predicts metabolite abundance between cases and controls should not be influenced by confounding exposures. Even more directly, Mendelian randomization (MR) studies have recently attracted much attention, as they evaluate the genetic effect of modifiable exposure, such as metabolites, on an outcome (15–17). MR tests the hypothesis that there is a correlation between the effect size of genetic variants on metabolite levels and the effect size of those variants on disease. There are assumptions about MR genetic instruments, including that they must be associated with the exposure trait, that they are not associated with the outcome trait via another mechanism, and that the association with the outcome occurs only through exposure. Rejection of the null that there is no such correlation, after correction for possible influences of pleiotropy, provides strong evidence that the metabolite is causally implicated in disease risk or prevention.

To date, several dozen studies have used MR to evaluate the role of PUFAs in a variety of diseases. We and others used more than two dozen genetic instruments associated with omega-3 fatty acids (ω_3_FA) at genome-wide significance levels, to establish that ω_3_FA protected against the onset of inflammatory bowel disease (15, 18, 19). Since a high ratio of ω_6_ to ω_3_ was found to elevate risk, this analysis provided further evidence for the anti-inflammatory benefits of ω_3_FA, and this conclusion also applies to protection against atopic dermatitis (20) and severe COVID-19 (21). Similarly, MR has shown that ALA, but not EPA or DHA, is protective against ischemic stroke (22), as well as certain types of cancer (23, 24) (esophageal, colorectal, and lung) but not others (reproductive, nervous system, or blood), and has also questioned whether ω_3_FA protect against major depression, schizophrenia, or anorexia nervosa (25–27) or various cardiovascular disease endpoints (28). Conversely, it has also been suggested that ω_3_FA may be a risk factor for epilepsy (29). Given widespread beliefs that ω_3_FA provides protection against many diseases, there is a pressing need for a well-powered genetic evaluation of causality.

Large-scale biobank studies provide an opportunity to perform such evaluations in a relatively unbiased manner (30). Here, we utilize the UK Biobank cohort study of over 500,000 United Kingdom residents to evaluate PUFA-disease associations with over 1,300 diseases. We restrict our analyses to white British (European ancestry) subjects to avoid complications of population stratification, and after quality control, we identified over 190,000 individuals with ω_3_FA, ω_6_FA, and DHA abundance data and whole genome genotypes and used four approaches to evaluate metabolite-disease associations: direct measurements, polygenic risk score (PRS) association, MR, and PRS-by-obesity interactions. Hereafter, we use PRS-d to refer to the polygenic risk score for a disease and PGS-m to refer to the polygenic score for a metabolite. We find that the three PUFA measures are all overwhelmingly protective for upward of 170 different conditions but with differing enrichments for respiratory, endocrine/metabolic, and gastroenterological diseases. Furthermore, genetic mediation of protection shows unexpectedly high modulation by obesity status, which sometimes overwhelms the genetics and sometimes exacerbates it differently for waist-to-hip ratio (WHR) and body mass index (BMI) stratification. We interpret these findings in the context of canalization of the evolved risk of disease (31, 32).

## 2 Materials and methods

### 2.1 Study cohort

Participants were ascertained from the UK Biobank (UKB), a cohort study of approximately 500,000 individuals from the United Kingdom and multiple ancestries, although predominantly European ancestry. For this analysis, previously imputed genotype and phenotype data were utilized. Analysis of the UKB data was performed under the approval of project number 17984.

The imputed genotype data, released in May 2017, covering 96 million variants, were extracted and filtered for bi-allelic variants, imputation score > 0.9, MAF > 1%, Hardy–Weinberg equilibrium value of *p* >10^−10^, and missing rate < 5%. This resulted in approximately 8 million SNPs observed in 487,409 individuals.

Individual-level phenotype data, covering clinical outcomes, quantitative measurements, and touch-screen responses, were extracted from the UKB Data Showcase in November 2023. We restricted the analyses to unrelated (no kinship found; field ID: 22021), white British (field ID: 22006) individuals whose genetically assessed sex (field ID: 22001) was the same as their self-reported sex (field ID: 31) and individuals who do not have sex chromosome aneuploidy (field ID: 22019). In addition, we restricted the cohort analyses to individuals having phenotypic information for the selected covariates, such as age (field ID: 21022), sex (field ID: 31), first 10 PCs (field ID: 22009 a{1:10}), BMI (field ID: 21001), waist circumference (field ID: 48), and hip circumference (field ID: 49). Individuals who had elected to withdraw from the UKB study at the time of data accession were excluded. This resulted in 276,169 individuals after the imputed genotype and phenotype data quality control (QC). A preliminary analysis was also performed on 121,643 members of the cohort whose metabolite data were available in 2022 and that included siblings and other individuals with kinship.

### 2.2 UKB fatty acid metabolite levels

There are approximately 280,000 individuals in the UKB with reported metabolite levels after the September 2023 data release. Of these, we analyzed only individuals with genotypes, passing the sample QC filters, which resulted in a sample size of 101,793. Metabolite levels were extracted for ω_3_FA (field ID: 23444), ω_6_FA (field ID: 23445), and DHA (field ID: 23450). In addition, the metabolite level QC flags were extracted for ω_3_FA (field ID: 23744), ω_6_FA (field ID: 23745), and DHA (field ID: 23750). If a QC flag field for a given metabolite reported a warning (e.g., unknown contamination), that metabolite level for the individual was removed. We had 155,486, 155,494, and 155,476 individuals passing QC for ω_3_FA, ω_6_FA, and DHA, respectively. For individuals with more than one reporting of the metabolite level, the median of the levels was taken. A metabolite Z-score for the metabolite level was evaluated using the following formula: $z=\frac{x-\mu }{\sigma }$, where x is the median metabolite level for an individual, μ is the mean of the median metabolite levels in the sample, and σ is the standard deviation of the median metabolite level.

### 2.3 UKB disease cohorts

ICD-10 summary diagnosis codes were extracted from UKB to create case and control cohorts using the case inclusion and control exclusion criteria for each disease-specific phenotype code (phecode) using the phecode mappings^1^ (33). There were 1,755 potential disease endpoints to assess; however, only diseases with at least 50 cases were analyzed, which resulted in the evaluation of 1,254 diseases and disease sub-types.

### 2.4 Non-genetic associations of fatty acid metabolites and diseases

Logistic regression models were implemented to assess the association between each Z-score_Metabolite_ and disease phecode (case versus control). Age, sex, age^2^, and the first 10 global genotypic principal components were included in the model as covariates.


$$\begin{array}{l} \text{Disease status}\sim {\text{Zscore}}_{\text{Metabolite}}+\text{age}\\ +\text{sex}+{\text{age}}^{2}+{\text{PC}}_{1}+\ldots +{\text{PC}}_{10} \end{array}$$

### 2.5 Genetic associations of fatty acid metabolites and diseases

Supplementary Table S1 summarizes the details, including the sample size, population, and references for each GWAS summary statistic utilized. The Bayesian approach to PRS calculation, PRScs (34), was used, estimating posterior SNP effect sizes under continuous shrinkage (CS) priors for each GWAS summary statistic using the UK BioBank European linkage disequilibrium (LD) reference panel.^2^ The inferred posterior effect sizes were then used to generate PGS-m or PRS-d across chromosomes using PLINK’s score function and then summed for each individual (35). Logistic regression models were performed to assess the association between each scaled polygenic score for the metabolite (PGS-m) and disease phecode (case versus control). Age, sex, age^2^, and the first 10 global principal components were included in the model as covariates.


$$\text{Disease status}\sim {\text{PGS}}_{m}+\text{age}+\text{sex}+{\text{age}}^{2}+{\text{PC}}_{1}+\ldots +{\text{PC}}_{10}$$

### 2.6 Assessment of suggestive fatty acid metabolite—disease associations

A multiple testing value of p threshold of 6.65 × 10^−6^ (0.05/(1,254 (number of diseases)*(3 (PUFA metabolites)*2 (Z-score and PGS_Metabolite_)))) was applied for assessing the significance of the metabolite-disease non-genetic and genetic associations. A fatty acid metabolite-disease association was inferred if there were overlapping significant non-genetic and genetic associations with concordant directions of effect (OR > 1 = Risk or OR < 1 = Protective).

### 2.7 Evaluation of phenotypic differences across fatty acid metabolites

Concordant and discordant disease associations across the three metabolites were evaluated to identify shared diseases and diseases unique to each metabolite. A relative risk (RR) score for each disease group was computed for each metabolite to determine if the metabolites have distinct disease areas of association. The RR was computed by evaluating the number of significant diseases in a disease group ($N_{\text{significant diseases in group}}$), the total number of significant diseases ($N_{\text{significant diseases}}$), the total number of diseases in the group ($N_{\text{diseases in group}}$), and the total number of diseases ($N_{\text{total diseases}}$). The calculation is as follows:


$$\text{RR}=\frac{\frac{N_{\text{significant diseases in group}}}{N_{\text{significant diseases}}}}{\frac{N_{\text{diseases in group}}}{N_{\text{total diseases}}}}$$

Fisher’s exact test was used to develop a contingency table to evaluate the significance of the RR:


$$\left(\begin{array}{cc} N_{\text{significant diseases in group}} & \begin{array}{l} N_{\text{significant diseases}}\\ -N_{\text{significant diseases in group}} \end{array}\\ N_{\text{diseases in group}} & \begin{array}{l} N_{\text{total diseases}}\\ -N_{\text{diseases in group}} \end{array} \end{array}\right)$$

A multiple testing value of p threshold was applied to control 15 disease groups with at least 1 significant disease, such as a value of p threshold of 0.0033 (0.05/15).

### 2.8 MR evidence of suggestive fatty acid metabolite—disease associations

There were 184 unique significant diseases across the 3 fatty acid metabolites. Each of the 184 diseases was manually mapped by its name to a well-powered GWAS summary statistic available in the OpenGWAS database (36). These diseases were mapped based on having an exact match as per the phecode name or by having a synonymous or close disease term. Information on the exposure (metabolite) and outcome (disease) GWAS summary statistics (e.g., trait name, sample size, etc.) used for performing MR can be found in Supplementary Table S1. Two-sample Mendelian randomization (17) was performed to assess the causal association between the three metabolites as exposures and the 184 diseases as outcomes. The same MR and the instrumental variable outlier detection and removal approaches described in our previous study were applied (15). A nominally significant *p*-value threshold of 0.05 was applied using the inverse variance weighted (IVW) MR method to determine significant associations (16). We also applied the weighted median test and highlighted associations with both IVW and weighted median signal in the resulting figure. Because the suggestive fatty acid metabolite-disease associations were all protective, we only assessed MR associations with OR < 1. The OR of the suggestive and causal associations for each metabolite was then compared.

### 2.9 Canalization of PUFA metabolites on disease

To assess the dependency of PUFA metabolites on disease, we adapted an approach defined by Nagpal et al. (32), to assess potential canalizing effects. Canalization of the PUFA metabolites on disease was evaluated for a few metabolite-disease associations with metabolite, metabolite PGS, and MR support. The PRS-d for cholelithiasis, major depressive disorder, and diabetic retinopathy was computed using the same approach as the metabolites. The GWAS summary statistics from which the variant weights were derived are presented in Supplementary Table S1. These diseases were selected as they had not only non-genetic and genetic support for at least one of the metabolites but also MR support. Specifically, we evaluated canalization for the following associations: cholelithiasis and ω_3_FA, major depressive disorder and ω_3_FA, cholelithiasis and ω_6_FA, diabetic retinopathy and ω_6_FA, cholelithiasis and DHA, and major depressive disorder and DHA. To evaluate canalization of the PUFA metabolites on disease, the cohort was first dichotomized by the Z-score_Metabolite_, whereby high (Z-score_Metabolite_ ≥ μ_Metabolite_) and low groups (Z-score_Metabolite_ < μ_Metabolite_) were defined by being above or below the mean, respectively. Next, the prevalence for the disease was computed for 100 bins of the PRS-d for the high and low cohorts. We then evaluated the PRS-d percentile versus the prevalence of the disease for high/low PUFA metabolite.

### 2.10 Interaction between PUFAs and body weight measurements on disease

BMI (field id: 21001), waist circumference (field id: 48), and hip circumference (field id: 49) were extracted from the UKB. For individuals with more than one reporting the body weight measurement, the median of the available measurements was taken. Next, the waist-to-hip ratio was computed using the median values for waist circumference and hip circumference (i.e., waist circumference/hip circumference). A Z-score was evaluated for BMI and waist-to-hip ratio using the following formula: $Z-{\text{score}}_{\text{body weight measurement}}=\frac{x-\mu }{\sigma }$, where x is the median measurement, μ is the mean of the median measurement, and σ is the standard deviation of the measurement. To evaluate the genetic and non-genetic interactions of the suggestive metabolite-disease associations, the associations were then subjected to logistic regression models, assessing the interaction between the genetic (PGS_m_) and exposure (Z-score_Metabolite_) effects with the two body weight measurements, BMI and waist-to-hip ratio on the outcome disease. The constructed models were as follows:


$$\begin{array}{l} \text{Disease status}\sim {\text{PGS}}_{m}+Z-{\text{score}}_{\text{Body weight measurement}}\\ +{\text{PGS}}_{m}*Z-{\text{score}}_{\text{Body weight measurement}}\\ +\text{age}+\text{sex}+{\text{age}}^{2}+{\text{PC}}_{1}+\ldots +{\text{PC}}_{10} \end{array}$$

$$\begin{array}{l} \text{Disease status}\sim Z-{\text{score}}_{\text{Metabolite}}\\ +Z-{\text{score}}_{\text{Body weight measurement}}+Z-{\text{score}}_{\text{Metabolite}}*Z\\ -{\text{score}}_{\text{Body weight measurement}}+\text{age}+\text{sex}+{\text{age}}^{2}+{\text{PC}}_{1}+\ldots +{\text{PC}}_{10} \end{array}$$

In addition, we ran the same models using obesity status for BMI and WHR, instead of the Z-score_Body weight measurement_. The cohort was divided into two cohorts for each body weight measurement, BMI and WHR, defining obese and non-obese by standard cutoff values. The BMI_Obese_ group had individuals with a BMI > 30, while the BMI_Non-obese_ group comprised individuals with a BMI ≤ 30 (37). The WHR_Obese_ group had individuals with a WHR > 0.85 for women and WHR > 0.95 for men, and the WHR_Non-obese_ group had individuals with a WHR ≤ 0.85 for women and a WHR ≤ 0.95 for men (38).

### 2.11 Canalization of body weight measurements and obesity on PUFA metabolites and the impact of it on disease

A set of disease-metabolite associations identified by the aforementioned interaction models, assessing PUFA metabolites and weight measurements on disease, were further used to assess the potential events of canalization of the body weight measurements on PUFA metabolites and the impact of it on disease. Specifically, type 2 diabetes and ω_3_ fatty acids, obstructive chronic bronchitis and ω_3_FA, type 2 diabetes and DHA, chronic obstructive bronchitis and DHA, and diaphragmatic hernia and DHA. The cohort was divided into two sets for each body weight measurement, with BMI and WHR defined as obese and non-obese using standard cutoffs reported in the previous section. The prevalence for the disease was computed for 100 bins of the PGS_Metabolite_ for the obese and non-obese body weight measurement cohorts. To quantify canalization, we first computed delta observed, which is the difference between the extreme 2% right and left tail differences of the obese and non-obese groups. We then computed delta expected, which is the difference between the right and left tail differences, simulating the disease prevalence for 10 iterations and assuming equal variance in each percentile group. These two values were then used to compute delta departure, a scaled estimate of the departure between observed delta and expected delta, a measure of canalization. More information on this approach is explained in the study by Nagpal et al. (32).

## 3 Results

### 3.1 Direct clinical and polygenic PUFA—disease associations

Assessment of the direct association of three PUFAs with over 1,200 prevalent diseases or clinical conditions revealed that DHA was the most associated, followed by ω6FA and ω_3_FA. A total of 170 significant (*p*-value <6.65 × 10^−6^) disease associations with ω_3_FA were observed, of which 165 were protective associations (OR < 1) and 5 were risk associations (OR > 1). The five risk associations for ω_3_FAs were uterine leiomyoma (OR = 1.08; 95% CI: 1.05–1.11; *p*-value = 1.07 × 10^−6^), hyperlipidemia (OR = 1.10; 95% CI: 1.07–1.14; *p*-value = 8.19 × 10^−11^), hypercholesterolemia (OR = 1.11; 95% CI: 1.09–1.13; *p*-value = 1.47 × 10^−44^), mixed hyperlipidemia (OR = 1.36; 95% CI: 1.19–1.54; value of *p* = 1.91 × 10^−6^), and gout (OR = 1.13; 95% CI: 1.09–1.18; value of *p* = 7.97 × 10^−10^). Similarly, 236 significant disease associations were observed for ω_6_FAs, all of which were protective. There were 285 significant disease associations with DHA, of which 284 were protective associations and 1 was risk association, for contracture of palmar fascia [Dupuytren’s disease] (OR = 1.12; 95% CI: 1.07–1.18; *p*-value = 3.25 × 10^−7^). The summary statistics for the significant non-genetic disease associations for ω_3_FAs, ω_6_Fas, and DHA are demonstrated in the “Disease-Metabolite level associations” tab on https://pufa.biosci.gatech.edu and Supplementary Table S2. Notably, 22.4% of the significant DHA non-genetic protective disease associations did not overlap with those detected with ω_3_FAs or ω_6_FA, while ω_6_FA and ω_3_FA only yielded 14.7 and 0.6% of unique disease associations, respectively.

Next, we evaluated whether the PGS for each metabolite was associated with the diseases. The results were strongly concordant with the direct clinical assessments, but since only ~50% of each metabolite level is explained by the PGS, fewer associations were observed. There were 33 significant (*p*-value <6.65 × 10^−6^) diseases for ω_3_FA, of which 31 were protective associations (OR < 1) and 2 were risk associations (OR > 1). The two risk associations for ω_3_FA genetics were hyperlipidemia (OR = 1.13; 95% CI: 1.10–1.17; *p*-value = 6.5 × 10^−16^) and hypercholesterolemia (OR = 1.13; 95% CI: 1.11–1.14; *p*-value = 1.5 × 10^−65^). There were 50 significant diseases for ω_6_FA, of which 48 were protective associations and 2 were risk associations. The two risk associations were also hyperlipidemia (OR = 1.07; 95% CI: 1.04–1.05; value of *p* = 2.0 × 10^−6^) and hypercholesterolemia (OR = 1.10; 95% CI: 1.08–1.11; *p*-value = 9.0 × 10^−42^). Additionally, 139 significant disease associations were detected for DHA, of which 138 were protective associations and 1 was risk association, for prostate cancer (OR = 1.06; 95% CI: 1.04–1.09; *p*-value = 2.3 × 10^−6^). The summary statistics for the significant genetic disease associations for ω_3_FA, ω_6_FA, and DHA are presented in https://pufa.biosci.gatech.edu on the “Disease-Metabolite PGS associations” tab and Supplementary Table S3. With the non-genetic associations, 58.5% of the DHA PGS associations were distinct from those observed with ω_3_FA and ω_6_FA. In contrast, 99.3% of the ω_3_FA genetic disease associations overlapped with either ω_6_FAs or DHA, while 4.8% of ω_6_FA genetic disease associations were unique to ω_6_FA levels.

**Figure 1:**
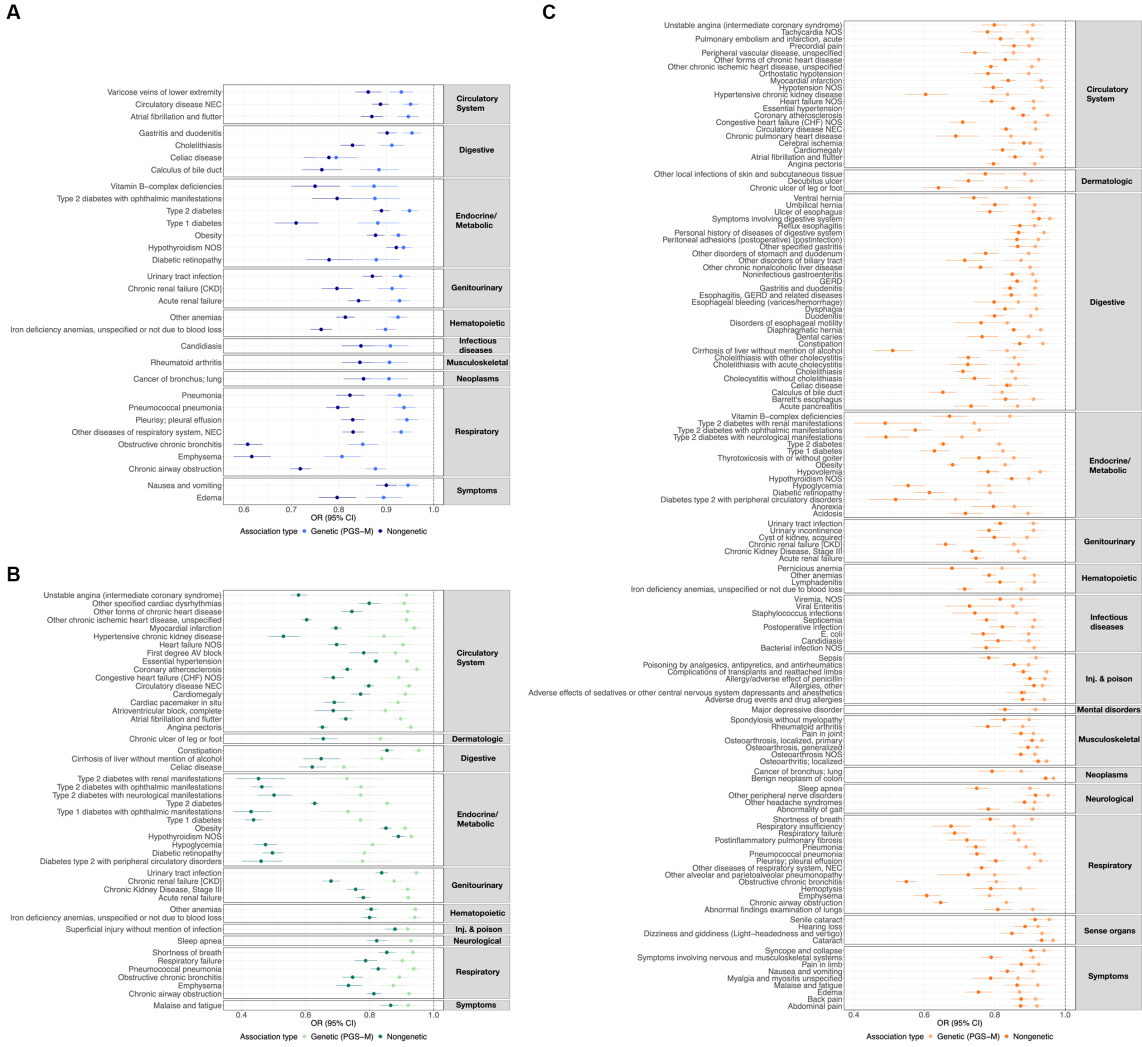
ω_3_FA, ω_6_FA, and DHA concordant disease associations. OR and 95% CI representing the genetic (PGS-m) and direct clinically significant protective disease associations for **(A)** ω_3_FAs, **(B)** ω_6_FAs, and **(C)** DHA.

**Figure 2:**
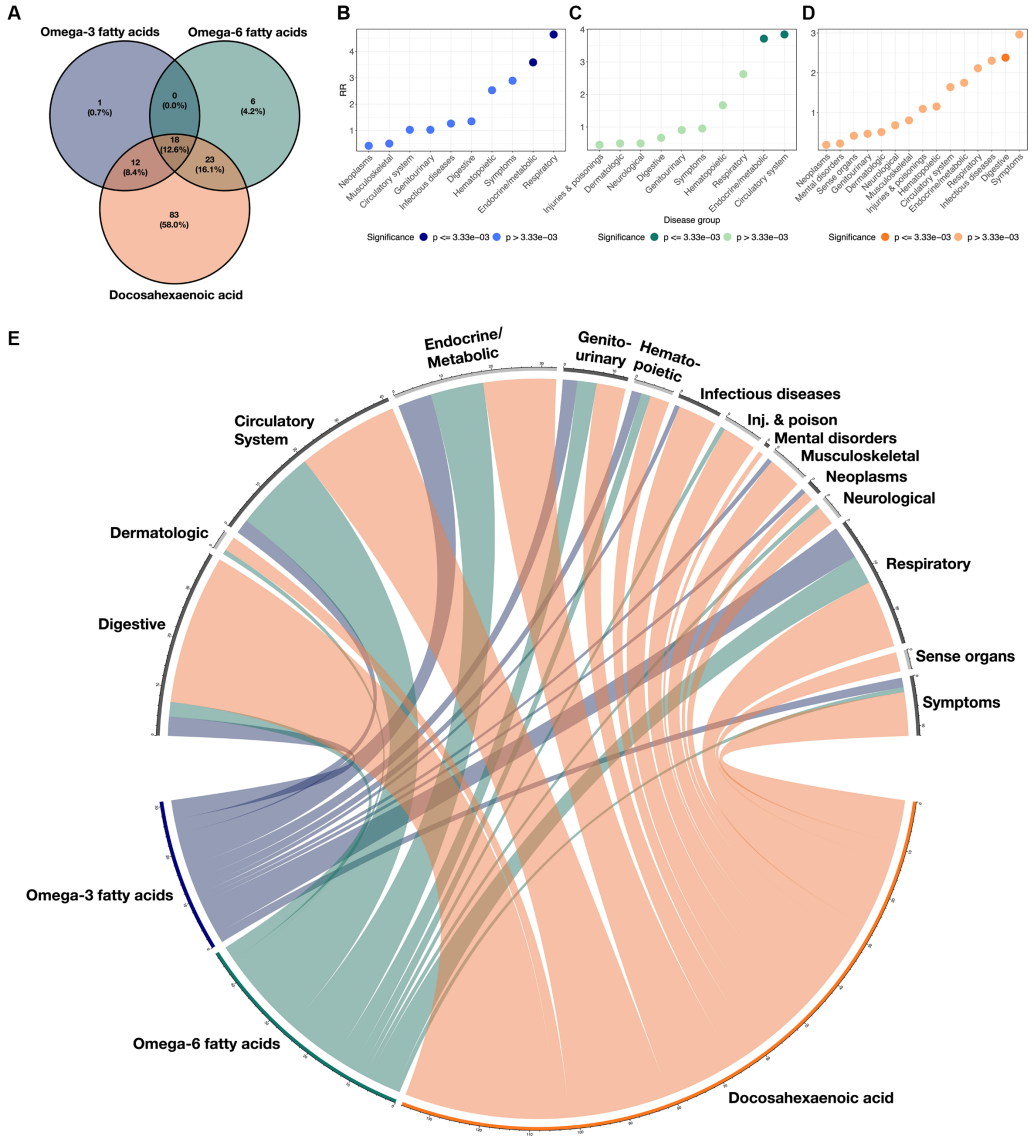
Overlaps of disease classes with ω_3_FAs, ω_6_Fas, and DHA. **(A)** Venn diagram demonstrating the overlap of significant protective clinical and genetic disease associations across ω_3_FAs, ω_6_Fas, and DHA. **(B–D)** RR implying enrichment for each indicated disease group colored by significance. **(E)** Circos plot representing the enrichment of each disease group across the three PUFA metabolites. The width of each ribbon is proportional to the number of significant associations between each disease class and metabolite.

Additionally, we characterized the directional similarity of protective disease associations, which is significant for both the clinical and genetic assessments. Figure 1A shows the 31, 47, and 136 cases for ω_3_FAs, ω_6_FAs, and DHA, respectively. Despite the large number of ω_3_FA and ω_6_FA indications, DHA has an additional 58% of its associations as distinct. Strong overlap, as shown in Figure 2A, is expected since the correlation of the three PUFA polygenic scores is greater than 0.85 for ω_3_FAs and DHA and approximately 0.4 for ω_6_FAs.

Enrichment for associations in each of the 15 disease groups was quantified for each metabolite by computing a relative risk (RR) score defined as the ratio of the proportion of significant diseases due to the group, to the proportion of each group in the total number of diseases (see Methods section). These scores are presented in Figures 2B–D for each metabolite, and the cross-correlations are presented as a circos plot in Figure 2E. Notably, ω_3_FAs were enriched for endocrine/metabolic and respiratory conditions, and DHA was enriched for digestive diseases, while ω_6_FAs were more likely to be attributed to diseases of the circulatory and endocrine/metabolite systems. Overall, neoplasms and musculoskeletal disorders were under-represented, as they were not significantly impacted by PUFA metabolites. While mental health disorders are also generally not associated, below we do explore the impacts of the metabolites on major depression.

### 3.2 Body weight by PUFA interaction influencing disease prevalence

For each of the diseases associated with both metabolite and PGS-m measures, we next asked whether these associations are a function of two measures of body weight and obesity cutoffs, by fitting logistic regression models and evaluating the significance of the interaction terms. These results are presented in Supplementary Figure S1, where we show the diseases for each metabolite that were nominally significant from the interaction model and also significant for metabolite and PGS-m measures. BMI is the ratio of mass-to-zheight-squared and is thought to relate more to eating behavior since GWAS findings are enriched for neuronally expressed genes, whereas WHR is more likely to reflect metabolic gene function (39–42). The two measures are highly correlated, but a much higher proportion of women is characterized as obese using standard WHR cutoffs (Supplementary Figure S2). Nevertheless, significant interaction effects are overall quite similar for the two body weight measures when comparing metabolite and PGS-m measures.

Although ω_3_FAs have the fewest associations with disease, they engage in the greatest proportion of interactions, approximately one-third for both the metabolite and the ω_3_FA PGS as compared with less than a quarter for ω_6_FAs or DHA. In addition, it is notable that DHA shows more interaction effects with the genetic risk than with the metabolite for diseases of the circulatory system, suggesting, for example, that the protective effect of DHA on peripheral vascular disease is predicted to be a function of obesity from the genetics, but this interaction is offset by dietary intake of DHA. Conversely, endocrine/metabolic disease, particularly type 2 diabetes, shows the opposite trend, showing a highly significant interaction between WHR or BMI and DHA that is less obvious for the genetic component of the metabolite.

Considering specific interaction effects, both type 1 and type 2 diabetes seem to increase in ω_3_ fatty acid protective effect as weight (measured as either BMI or WHR) increases, but type 1 diabetes is not affected by interactions between body weight and DHA. Interestingly, hypoglycemia has strong interaction effects with ω_3_FA genetics. The only statistically strong interactions involving ω_6_FAs are with essential hypertension, and with diabetic retinopathy, explored in more detail in the next section. DHA and ω_3_FAs also show a series of genetic and direct metabolite interactions between obesity and multiple modes of respiratory disease, including chronic bronchitis, airway obstruction, and emphysema. A noteworthy interaction involving DHA and body weight is with diaphragmatic hernia. Each of these cases illustrates the importance of considering PUFA associations with disease as a function of obesity.

### 3.3 Mendelian randomization

**Figure 3:**
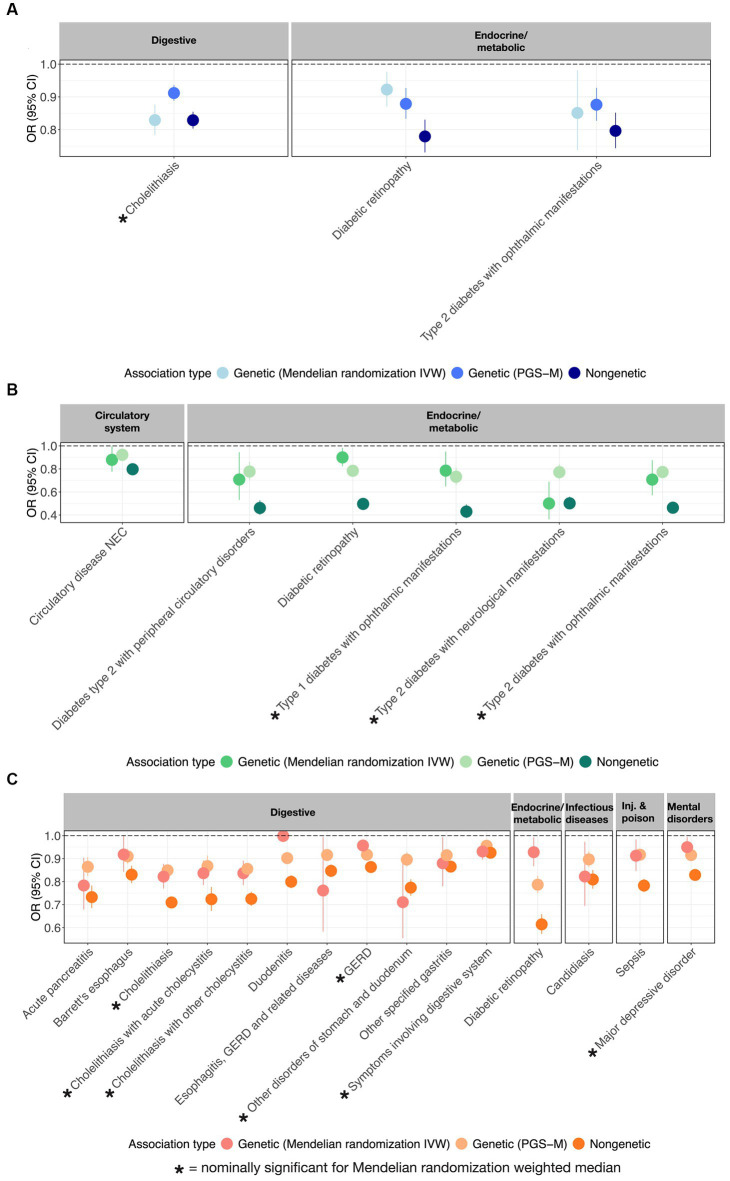
MR-supported ω_3_FA, ω_6_FA, and DHA disease associations. OR and 95% CI representing from left to right the genetic (MR), polygenic (PGS-m), and clinical protective disease associations supported by all three assessments for **(A)** ω_3_FAs, **(B)** ω_6_FAs, and **(C)** DHA.

Further evidence that PUFAs are causally protective against multiple diseases was sought by performing Mendelian randomization analyses using the inverse variance method (IVW) and the weighted median methods (14–16). Figure 3 illustrates the odds ratio per standard deviation of the metabolite, the PGS, or the mediating effect of the PGS, for each of 3 ω_3_FAs, 6 ω_6_FAs, and 15 DHA associations that also have nominally significant MR results. These are also summarized in the “Metabolite~Disease Mendelian randomization associations” tab on https://pufa.biosci.gatech.edu, which includes the summary statistics across five MR methods that have nominal significance (*p*-value <0.05). In addition, these results are also presented in Supplementary Table S4. Notably, in all cases, the inferred effect is protective. Cholelithiasis (gallstones) is causally implicated for ω_3_FAs and DHA. Major depression is specific to DHA, and ω_6_FAs are uniquely likely to be causal for neurological and ophthalmic complications of both types of diabetes, and (like DHA) with diabetic retinopathy. In contrast, DHA is multiply connected with gastric diseases, with implications for dietary associations with gut health.

### 3.4 Interaction between PRS for disease and PUFA levels modulates disease risk

**Figure 4:**
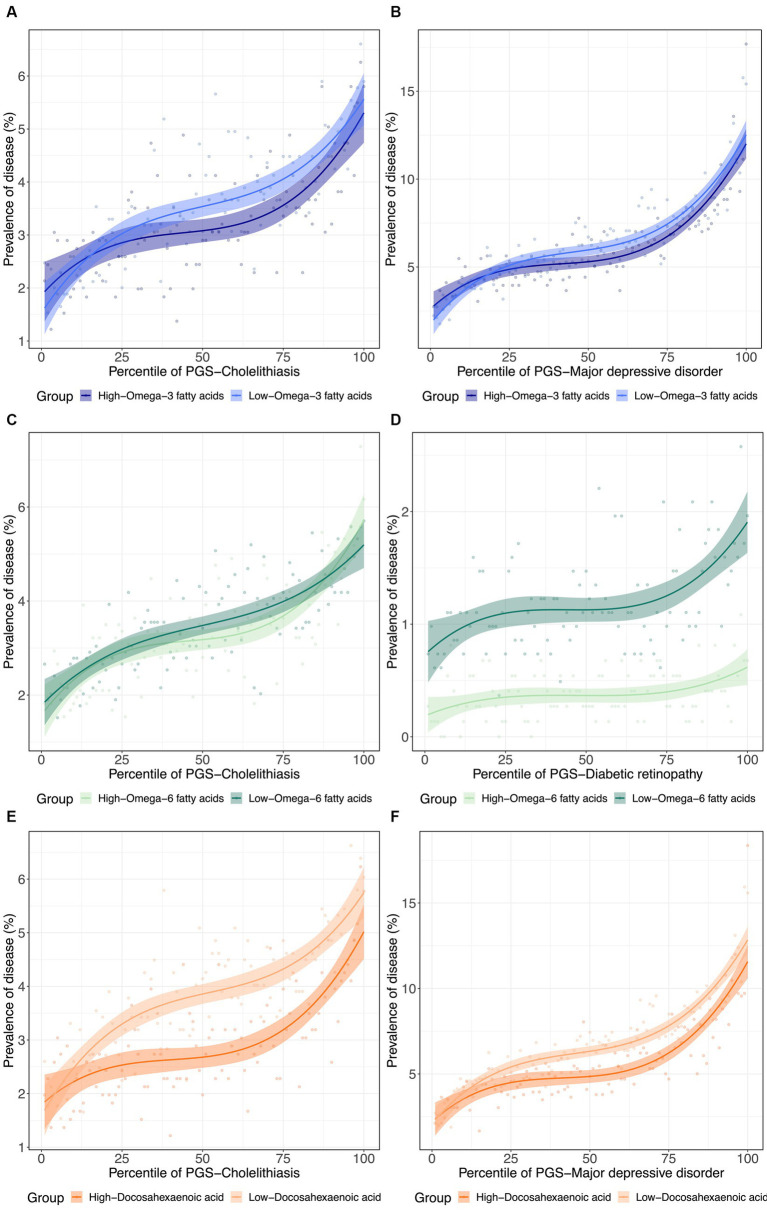
(De)canalization of polygenic risk for disease *via* PUFA dysregulation. **(A)** Prevalence of cholelithiasis versus PRS_Cholelithiasis_, dichotomized by low versus high $Z-{\text{score}}_{\omega_{3}\;\text{fatty acids}}$. **(B)** Prevalence of major depressive disorder versus PRS_Major depressive disorder_, dichotomized by low versus high $Z-{\text{score}}_{\omega_{3}\;\text{fatty acids}}$. **(C)** Prevalence of Cholelithiasis versus PRS_Cholelithiasis_, dichotomized by low versus high $Z-{\text{score}}_{\omega_{6}\;\text{fatty acids}}$. **(D)** Prevalence of diabetic retinopathy versus PRS_Diabetic retinopathy_, dichotomized by low versus high $Z-{\text{score}}_{\omega_{6}\;\text{fatty acids}}$. **(E)** Prevalence of cholelithiasis versus PRS_Cholelithiasis_, dichotomized by low versus high $Z-{\text{score}}_{\text{DHA}}$. **(F)** Prevalence of major depressive disorder versus PRS_Major depressive disorder_, dichotomized by low versus high $Z-{\text{score}}_{\text{DHA}}$.

To further visualize the nature of the metabolite-PGS by body weight interactions reported above, we next plotted the dependency of disease prevalence as a function of the PRS-d in individuals in the top and bottom halves of the distribution of the metabolite, as shown in Figure 4 as contrasting results. Notably, PRS_Cholelithiasis_, PRS_Major depressive disorder_, and PRS_Diabetic retinopathy_, had *p*-values of 5.2 × 10^−160^, 0, and 6.9 × 10^−28^, respectively, evaluating the PRS-d performance on predicting the disease via logistic regression models with DHA that include covariates for age, sex, and 10 genotypic PCs. For cholelithiasis, low ω_3_FA, or even more strikingly DHA, is a particularly adverse risk factor for intermediate levels of polygenic susceptibility. For major depression, the disease prevalence also becomes more similar for individuals with low DHA and the highest polygenic risk of depression. These two phenomena can be regarded as examples of canalization of disease since higher risk does not yield the expected degree of increased prevalence. For diabetic retinopathy, high ω_6_FA is protective essentially, regardless of polygenic risk, but the genetic influence is strong in individuals with low ω_6_FA.

### 3.5 Obesity modifies the influence of PUFAs on disease risk

**Figure 5:**
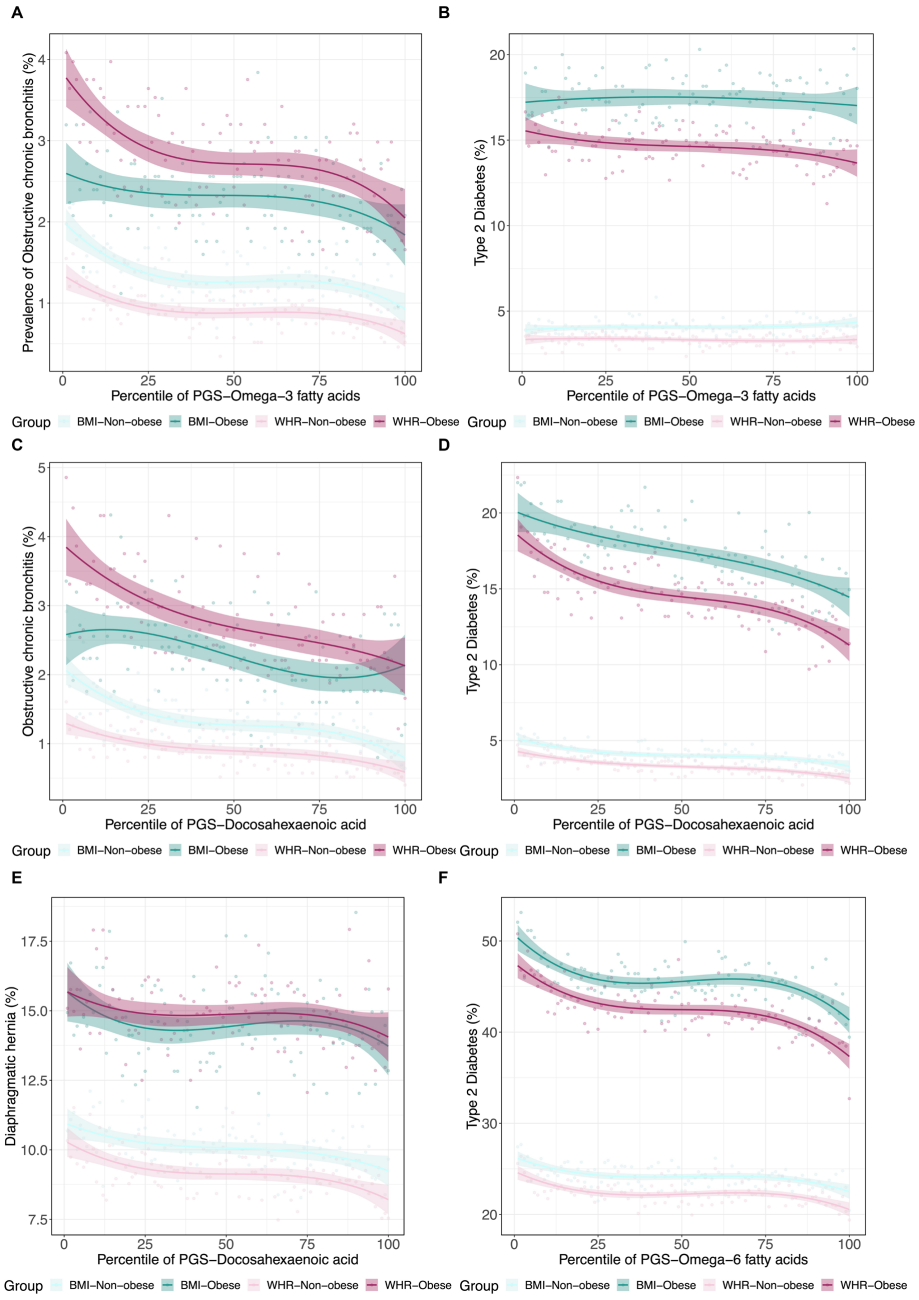
Canalization of polygenic risk for PUFAs in the context of obesity. **(A)** Prevalence of type 2 diabetes versus ${\text{PGS}}_{\omega_{3}\;\text{fatty acids}}$ in 4 groups: obese BMI, non-obese BMI, obese WHR, and non-obese WHR. **(B)** Prevalence of obstructive chronic bronchitis versus ${\text{PGS}}_{\omega_{3}\;\text{fatty acids}}$ in the four groups. **(C)** Prevalence of type 2 diabetes versus ${\text{PGS}}_{\text{DHA}}$. **(D)** Prevalence of obstructive chronic bronchitis versus ${\text{PGS}}_{\text{DHA}}$**. (E)** Prevalence of diaphragmatic hernia versus ${\text{PGS}}_{\text{DHA}}$. **(F)** Prevalence of essential hypertension versus ${\text{PGS}}_{\omega_{3}\;\text{fatty acids}}$ in the four groups.

Since obesity is both a metabolic and psychological disease, and to some extent, PUFA levels reflect dietary consumption, we next decided to investigate whether PUFA impacts on disease are modified in obese individuals as defined by WHR and BMI. Using a similar strategy, we further assessed the interactions between metabolite PGS and disease prevalence as a function of a binary classification denoting obesity status for WHR or BMI. Some representative examples are shown in Figure 5. In each case, obese individuals have two-fold to four-fold higher rates of disease, but the genetic predisposition for the fatty acid has different effects. For example, the ω_3_FA PGS protection against chronic bronchitis is almost completely absent in obese BMI individuals but exacerbated in WHR obesity. This situation is inverted for diaphragmatic hernia since the DHA PGS is only a risk factor in non-obese individuals as judged by WHR. Type 2 diabetes clearly illustrates a case where DHA but not ω_3_FAs is a whole influence disease risk. For essential hypertension, almost half of all obese individuals suffer from the condition, but higher levels of DHA seem to be even more protective in the WHR-obese group, though BMI also has an unexpectedly high dependency on the PGS.

To quantify interaction effects on this framework, we computed the delta departure for each metabolite and body weight (obese/non-obese) pair across the set of significant disease associations. Delta departure is the difference between observed and expected deviations in disease prevalence at the bottom and top two percentiles of the polygenic score for each disease (32). Because we are assessing protective associations, a positive delta departure implies that the two curves for the obese and non-obese groups show less deviation than expected (which can be interpreted as canalization) while a negative delta departure represents greater deviation than expected (which can be interpreted as decanalization). Full documentation of the delta observed, delta expected, and delta departure is presented in Supplementary Figures S3–S8, and individual cases can be viewed on our RShiny web application, https://pufa.biosci.gatech.edu. In addition, canalization results are presented in Supplementary Table S5.

**Figure 6:**
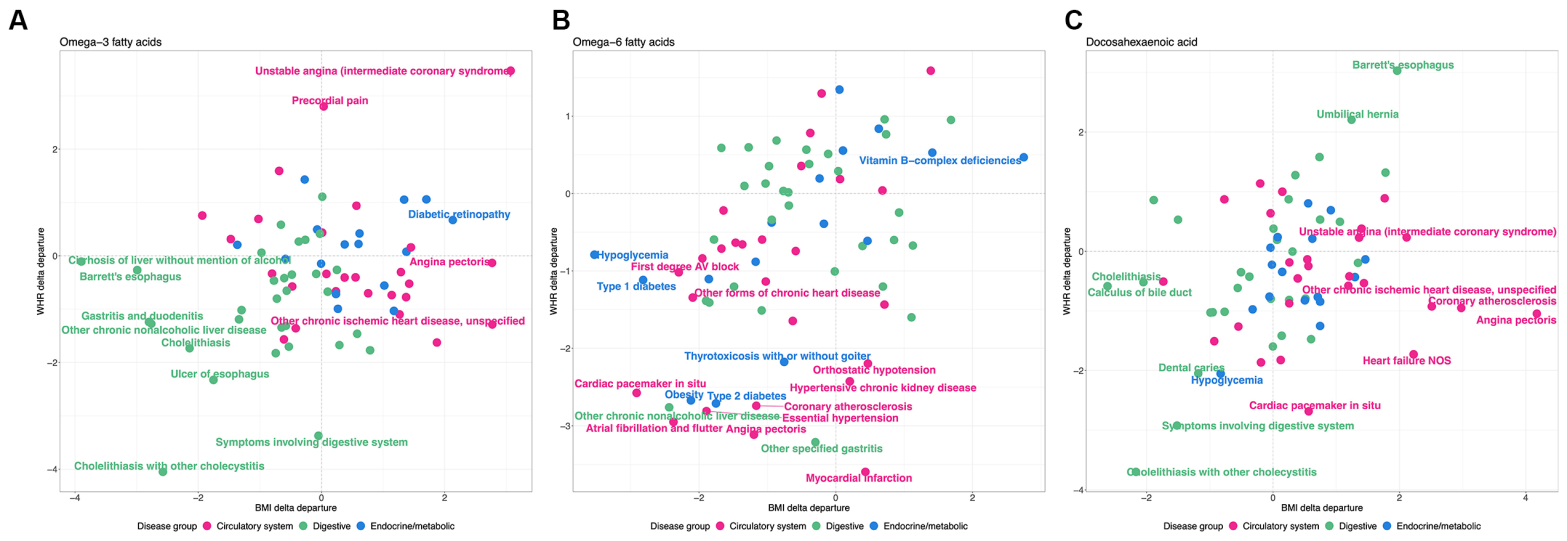
WHR and BMI affect disease classes differently. Each plot shows the delta differential, with BMI on the x-axis and WHR on the y-axis, for the observed versus expected deviation between the tails of the disease prevalence versus PGS-m percentile, for **(A)** ${\text{PGS}}_{\omega_{3}\;\text{fatty acids}}$, **(B)** ${\text{PGS}}_{\omega_{6}\;\text{fatty acids}}$, and **(C)** DHA, contrasting obese and non-obese groups. Green represents digestive system disease, blue represents endocrine and metabolic, and pink represents circulatory.

This analysis shows that there is a clear distinction between WHR-obesity and BMI-obesity in how they modify the genetic influence of PUFAs on disease prevalence in the UK Biobank. Notably, ω_3_FAs and DHA have some similar patterns in this analysis, which are significantly different from those observed with ω_6_FAs, as shown in Figure 6. Focusing just on diseases in the digestive group (green), endocrine/metabolic group (blue), or the circulatory system (pink), there are clear tendencies with respect to the delta departure measure. Specifically, digestive diseases such as cholelithiasis have negative measures for the ω_3_FAs and DHA PGS, implying enhancement of the genetic effect regardless of the mode of obesity. For ω_6_FAs, those are circulatory conditions (including essential hypertension and coronary atherosclerosis) that show this decanalization tendency, whereas such conditions are canalized in the high BMI group.

## 4 Discussion

Thousands of reports have addressed the association between PUFA and disease, but the literature is full of conflicting conclusions and uncertainties. Likely reasons for variable repeatability include heterogeneity in study design, participant attributes, analytical methods, and small sample biases. Very large population biobank studies overcome some of these difficulties, providing a relatively unbiased approach to epidemiological assessment in hundreds of thousands of individuals in whom hundreds of associations can be evaluated simultaneously. Using a tiered evidence approach starting with measurement of ω_3_FAs, ω_6_FAs, and DHA in just over 120,000 people reporting 1,350 different diseases or conditions, we confirm that ω_3_FAs generally and DHA specifically for the most part offer protection against a wide range of morbidities, as do ω_6_FAs, which may promote inflammation and metabolic disease. Then, incorporating genetics in the larger sample of almost 500,000 people reduces the number of significant associations, though it should be noted that polygenic scores only capture half of the variance for PUFA levels. Adding the stringent requirement for Mendelian randomization evidence reduces causal support to just 22 diseases or disease manifestations, which is still close to 2% of all of the assessments. All of our findings reported here replicate observations with a subset of the UKB cohort that included close relatives but only the first half of the metabolite release, with only minor deviations.

While writing up this study, we became aware of a similar PheWAS analysis of PUFA in the UK Biobank, which was reported by Zagkos et al. (30) in February 2023. They emphasized the protective influence of ω_3_FAs for cholelithiasis (gallstones) and obesity, contrasting with the risk for coronary heart disease posed by the ω_6_FAs linoleic acid. They included a multivariable Bayesian MR approach to tease apart the contributions of ω_6_FAs and ω_3_FAs and were able to independently replicate some findings in the FinnGen cohort (43). While we do observe many of their significant effects, our approach has uncovered additional associations and includes an extensive evaluation of polygenic influences across exposure groups. Major differences between the two studies include our use of the Bayesian PRScs (34) approach to genetic prediction of metabolites, performance of PRS-d association in the entire white British UK Biobank cohort (they exclude individuals contributing to metabolite measures), adoption of PRS-d from the OpenGWAS database (36), and evaluation of just 3 of the 8 PUFA measures which reduce the multiple comparison burden, all of which will have increased statistical power. We think that it is useful to have two parallel, independently conducted analyses and would argue that instances of disagreement deserve more detailed follow-up with additional large cohort studies.

Unique to our analysis is the quantification of obesity-by-metabolite interactions. Multivariable logistic regression models revealed that ω_3_FAs show fewer main effects overall than either ω_6_ fatty acids or DHA, which may be due to them being more likely to have modulated effects in obese individuals, who constitute over a quarter of the sample. It also suggests that genetic influences on DHA production that protect against peripheral vascular disease may be overcome by dietary intake, which conversely has a major influence on type 2 diabetes risk and pathology. Very strong associations of ω_3_FAs with hyperglycemia and ω_6_FAs with hypertension are also exacerbated by obesity. These findings imply that genetic effect sizes differ with weight gain, which will affect the performance of Mendelian randomization, which, in turn, likely underestimates causal inference in diverse cohorts.

Following our strategy for the detection of canalization of disease risk by contrasting the deviations at the tails of the prevalence—polygenic risk percentile curves between conditions, we adduce further evidence that obesity, itself mediated by PUFA, has a pervasive influence on how PUFA mediated disease. Given the proportion of the disease explained by PGS-m and the prevalence in two subsets of the data, we can contrast the observed deviations with those expected on the assumption that risk is constant across exposures. Since PUFAs are generally protective, large negative differences between obese and non-obese deviations in the prevalence between the high and low tails of risk provide evidence that genetic effects are enhanced in obese individuals. Positive differences imply the opposite, for example, if the genetic effects are suppressed, or if obesity itself overwhelms the genetic influence of the PUFAs. Across 178 diseases/conditions, two measures of obesity (BMI and WHR), and all three metabolites (1,068 comparisons), 66 were more than 2 standard deviations more deviant than expected and just 9 less deviant, implying considerably more decanalization and hence that low PFA exacerbate polygenic risk. Correspondingly, there were 177 cases of more than 1 standard deviation and 43 with less than expected deviation at this cutoff.

However, several patterns of disease class enrichment are highly significant. Notably, in Figure 6, digestive diseases tend to be decanalized (have enhanced genetic influence) for ω_3_FAs or DHA, whether obesity is measured by WHR or BMI, whereas circulatory and cardiovascular diseases only show this effect for WHR (and maybe canalized by high BMI). In addition, contrarily, the influence of ω_6_FAs on circulatory disease, as well as hypoglycemia and type 2 diabetes, is exacerbated for both classes of obesity. Evolutionary genetic theory suggests that canalization evolves under persistent stabilizing selection to buffer the effects of genetic and environmental perturbations. Decanalization implies the loss of protection afforded by PUFA in obese individuals, particularly those with low levels, and appears to be more consistently observed for WHR, which is a stronger indicator of metabolic perturbation than BMI. Our data thus imply that ω_3_FAs and ω_6_FAs have evolved different roles in protection against digestive, endocrine/metabolic, and circulatory diseases, and that contemporary obesity perturbs the protection disproportionately.

The implications of our findings for personalized medicine are debatable. Even if PUFAs are causally involved in diverse diseases, the heterogeneity of effects across disease classes calls for caution in therapeutic supplementation, despite the overwhelmingly protective nature of their associations. It is also not clear that if low levels of DHA promote disease, dietary supplementation after a certain age will be curative or preventative, but our data do support more clinical trials to evaluate this proposition. However, it also implies that obesity status needs to be included as a covariate in all such initiatives. For several diseases, PUFAs may be more protective in obese individuals than they are in the normal weight class, and in that group, it could be argued that disagreement between polygenic prediction and observed metabolite levels should be used to promote dietary supplementation. Thus, a person predicted genetically to have high ω_3_FA levels who does not have obesity, is likely to benefit from higher intake, but their decision should also be motivated by whether they are most concerned about gut, cardiovascular, endocrine, or even mental health.

The major limitations of this study are that few of the findings have been replicated with external datasets or PGS-m, and all of them are derived solely from European ancestry study participants. It will be particularly interesting to ascertain to what extent the findings also pertain to non-European ancestry groups and/or other countries with different dietary patterns, but this will require analysis of different biobank studies, such as All-of-Us. The results reported here may be specific to the white British participants in the UKB, who are themselves a biased representation of the total British population. Regarding replication, it should however be noted that there is a broad agreement between our results and those of the study by Zagkos et al. (30), generated with different PGS-m and confirmed in some instances in FinnGen (43). Where attempted, for example, for IBD and cholelithiasis, external replication has been strong (30). A third limitation is that the disease status in any population study may be strongly affected by disease comorbidity and the wide range of pharmaceutical and non-medical interventions that people take. Future research should address the likely implications for modification of PUFA influences on disease.

## Data availability statement

The original contributions presented in the study are included in the article/Supplementary material, further inquiries can be directed to the corresponding author.

## Ethics statement

Ethical approval was not required for the studies involving humans because all data was acquired from UK Biobank (approval number 17984). The studies were conducted in accordance with the local legislation and institutional requirements. Written informed consent for participation was not required from the participants or the participants’ legal guardians/next of kin in accordance with the national legislation and institutional requirements.

## Author contributions

CA: Conceptualization, Formal analysis, Investigation, Methodology, Visualization, Writing – original draft, Writing – review & editing. GG: Conceptualization, Funding acquisition, Supervision, Writing – original draft, Writing – review & editing.

## Footnotes

^1^ https://phewascatalog.org

^2^ https://www.dropbox.com/s/t9opx2ty6ucrpib/ldblk_ukbb_eur.tar.gz
