# Supplementary material for: Integrative polygenic analysis of the protective effects of fatty acid metabolism on disease as modified by obesity
Source: Front Nutr. 2024 Jan 15;10:1308622. doi: 10.3389/fnut.2023.1308622 (PMC10832455; doi:10.3389/fnut.2023.1308622)
Supplement: Supplementary file 6 [file Data_Sheet_1.DOCX]

Supplementary Material

# Supplementary Figures and Tables

## Supplementary Tables

(Excel file: “ST1_GWAS_Information.xlsx)

**Supplementary Table 1**. Meta information on the publicly available GWAS summary statistics utilized for computing PGS and Mendelian randomization.

(Excel file: “ST2_all_sig_nongenetic_metabolite_disease.xlsx)

**Supplementary Table 2**. Summary statistics for the significant metabolite Z-score disease associations.

(Excel file: “ST3_all_sig_genetic_metabolite_disease.xlsx)

**Supplementary Table 3**. Summary statistics for the significant PGS-m disease associations.

(Excel file: “ST4_MR_table.xlsx)

**Supplementary Table 4**. Summary statistics for the significant MR associations.

(Excel file: “ST5_Canalization_table.xlsx)

**Supplementary Table 5**. PGS-m x obesity canalization results.

## Supplementary Figures


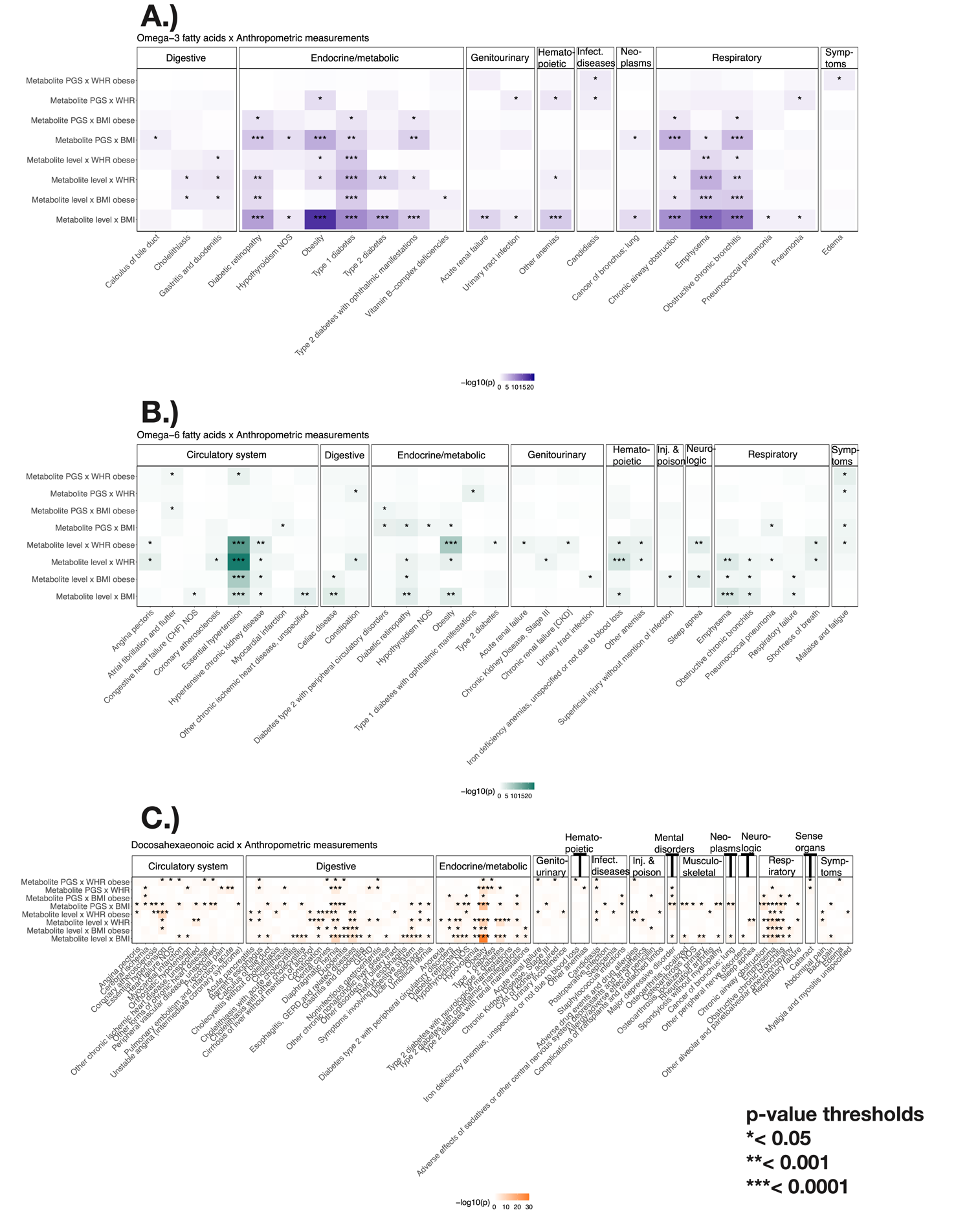


**Supplementary Figure 1.** PUFA Z-score_Metabolite_xObesity and PRS-MxObesity on suggestive disease associations. **(A)** ${Z-score}_{\omega_{3} fatty acids}$x BMI_Obese/Non-obese_ and ${Z-score}_{\omega_{3} fatty acids}$x WHR_Obese/Non-obese_ for all the suggestive disease associations for omega-3 fatty acids and $\mathrm{PGS}_{\omega_{3} fatty acids}$x BMI_Obese/Non-obese_ and $\mathrm{PGS}_{\omega_{3} fatty acids}$x WHR_Obese/Non-obese_ for all the suggestive disease associations for ω_3_ fatty acids. **(B)** ${Z-score}_{\omega_{6} fatty acids}$ x BMI and ${Z-score}_{\omega_{6} fatty acids}$ x WHR_Obese/Non-obese_ for all the suggestive disease associations for ω_6_ fatty acids and $\mathrm{PGS}_{\omega_{6} fatty acids}$x BMI and $\mathrm{PGS}_{\omega_{6} fatty acids}$ x WHR_Obese/Non-obese_ for all the suggestive disease associations for ω_6_ fatty acids. **(C)** ${Z-score}_{\mathrm{DHA}}$ x BMI_Obese/Non-obese_ and ${Z-score}_{\mathrm{DHA}}$x WHR_Obese/Non-obese_ for all the suggestive disease associations for DHA and $\mathrm{PGS}_{\mathrm{DHA}}$ x BMI and $\mathrm{PGS}_{\mathrm{DHA}}$x WHR_Obese/Non-obese_ for all the suggestive disease associations for DHA.Only the suggestive diseases with at least one star “*” were included in the figure.


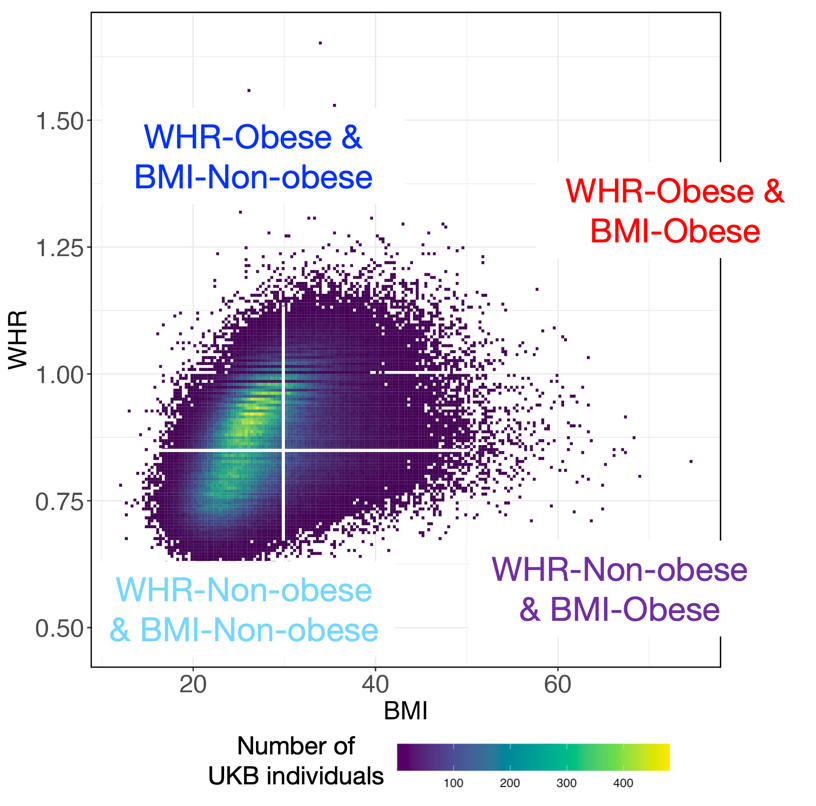


**Supplementary Figure 2.** BMI vs. WHR of individuals in the UKB. Increased enrichment regions (greater number of individuals) are colored with yellow while dark blue indicates less enrichment regions. The white horizontal line indicates the obesity threshold for WHR (average of male and female) and the white vertical line indicates the obesity (avg. male and female) threshold for BMI. This figure was generated using the white British UKB cohort, without removal of related individuals.
